# The Irish Immigrants’ Fever

**Published:** 1847-11

**Authors:** 


### RECORD OF MEDICAL SCIENCE.

### THE IRISH IMMIGRANTS' FEVER.

GROS ISLE, 33 miles below Quebec, August 27th, 1847.

To the EDITOR of the Boston Medical and Surgical Journal.

DEAR SIR,—Through the politeness of Mr. W. Stevenson, of Quebec, who runs a small steamboat for the government, I have been able to make a short visit to this quarantine station, and am now on my return to Quebec; or shall be, as soon as our little steamer takes on board the last of the convalescents from the fever hospitals, which I see waiting on the dock. Presuming that our brethren generally, throughout the United States, feel a lively interest in the disease which is prevailing here and up the St. Lawrence, even to our own country, I propose to give you a rapid and superficial sketch of what I have seen, and what I may hereafter see; although, as I have been travelling for more than two months, and seen but few medical journals, I do not know but others have already done for you what I am about to attempt. If so, please send on my letter to my colleagues of the Western Journal, at Louisville.

Gros Isle is one of the endless succession of beautiful islands which adorn this noble river, from Lake Ontario to the Gulf of St. Lawrence. Many of them consist of ancient *drift*, and have level surfaces which rise but a few feet above the water; but this is rocky and rugged, with heights of 80 or 100 feet, in its centre, and hence the name given it by the early French voyageurs. Its breadth is less than a mile, with a length of nearly two. The black birch, white cedar, and various kinds of pine, overshadow and partly obscure its stony surface.

The quarantine station is on the south-east or right hand side, which, in the approach, present three distinct groups of one story board sheds, some of which are mere cottages, but others from two to four hundred feet long. The lowest, or eastern group, is for the reception and temporary accommodation of immigrants in health; the next up the island, for the quarantine physician, and a small detachment of troops from the garrison at Quebec; the third, or western, more extensive than both the others, for the sick and their physicians, nurses, and a numerous body of carpenters, engaged in the erection of additional houses to receive the hundreds who are still lodged in tents and marquees. The buildings of each group are white-washed, and appear in pleasant contrast with the green slopes and tuberosities of the island, in their rear. The harbor in front presents several ships at anchor, and two or three steamboats, with a neat and nearly finished dock, projecting to a distance into the stream. When we were near the harbour, a gentleman, Mr. Patten, who resides in its neighborhood, and who had kindly directed my attention to different, objects on our little voyage, called my eye to an Irish immigrant ship, then passing us. On inspecting the group of passengers with a glass, I was surprised to find them so healthy in appearance; and when about to express myself to that effect, he discovered and announced that it was a ship from Bremen. Such is the difference between the German and Irish immigrants, in health and personal condition.

A distant view of Gros Isle suggests a new and busy colony, on a romantic shore; but a walk up the dock, which leads directly to the hospital sheds, most painfully dispels the pleasing illusion. As I approached them, the emaciated forms and haggard faces of convalescents, sauntering about, or crouched on the ground and rocks, or sitting underneath the eaves, and on the piles of boards to be used for coffins, impressively told what might be expected within. Conducted by Mr. Patten, I passed through them without stopping, till we reached the quarters of Dr. George M. Douglas, the health officer, who received me with much hospitality. I found him lame, from a kind of hospital gangrene or slough, which had attacked one of his feet, but, intent on his duties, he was bravely hopping about, and answering a hundred questions, or giving as many orders.

Taking me in his buggy, he drove to his office in the midst of the sick, where I was introduced to several of his assistants, They are chiefly young physicians of Quebec and other towns of Canada, employed by the government. One to whom I was introduced, although walking about, labored under fever; and yesterday I saw another at Quebec, who had returned in the same condition. The number of assistants to-day is nine. Since the first of June, twenty-one or twenty-two have been employed. All except Dr. Douglas, have experienced attacks of the fever, and three have died—one of whom was Dr. Frederick Cushing, formerly of the State of Maine. The exemption of Dr. Douglas is to be ascribed to his having already had the disease. After conversing awhile on its symptoms and treatment, Dr. Watt and Dr. Fenwick conducted me to their respective hospitals, embracing six or eight hundred patients, where I took such a *coup d'œil* of the sick as my limited time would permit, examining, with some attention, a considerable number in every stage of the disease. From a necessity which the Canadian government, up to this time, has been unable to avert, all the sheds and tents are crowded to such a degree, that one can scarcely turn round among the sick. Men, women and children, in all stages of the disease up to dissolution, are huddled together, and lying in the same foul and infectious clothes with which they started from Ireland; and which, no doubt they had worn, without change, for weeks or months before. The quarantine officers must not be blamed for this, since the means of classification and personal cleanliness are not within their reach. As to nursing, it is evidently in the lowest degree. Nearly all the nurses from Quebec have sickened, and the immigrants furnish but few from their own body. Their sympathies for each other are manifestly small—either never had an existence, or have perished under the combined influence of famine and filth. Examples of the well members of a family refusing to wait on the sick, are familiar to all the medical gentlemen; and a total indifference to the death of nearest relatives, is witnessed every day. Following their remains to the grave, or in any manner assisting in their interment, is not thought of. But one idea seems to be present with them, that of getting up the river. A man who had recovered, on being asked by some one, whether he was going to Montreal in the next steamboat, replied that he wished to do so, but was afraid his wife would not die in time. The family of a young woman who was ill, *sent* to know how she was before they started. On being expostulated with, they said it was not worth while to stay any longer, as she would no doubt die. Mr. Barter, the apothecary of the hospital, who is now by my side, going to Quebec on official business, confirms all that has been told me by others, and adds, as the result of his own observation throughout the summer, that the living seem more pleased than grieved by the death of their friends. My own limited opportunities suggest the same unwelcome conclusion; for I saw no aspect of sorrow; but a stolid indifference, or inquisitive gazing, at what might be passing around, both in the crowds of convalescents, and in patients not very ill, who lay in the midst of the dying. It is painful to record this testimony against human nature; but we ought to know to what depths of degradation large masses of people may be sunk by superstition, ignorance, bad legislation, famine and fever. The interests of political economy, religion and medicine, are equally involved in the contemplation of such revolting facts.

Quebec, August 28th.

Before and since my trip to Gros Isle, I have visited the Marine Hospital of this city (under the care of several of its most respectable physicians), where a great number of seamen are down with the fever, and near which there are extensive sheds, filled with sick immigrants. I have also been at the House of Correction, and in the Hotel Dieu, where I saw cases; and at the private hospital of Dr. James Douglas and Dr. Racey, in Beauport, a village three miles from the city, where I saw still more. Many of the cases I examined with care, and held conversations, more or less protracted, with a number of the medical attendants, among whom I may mention Dr. Morin, Dr. Racey, Dr. J. Douglas, Dr. Clark, and Dr. Fremont, whom I may unite with the physicians of Gros Isle, as the authors of what I am about to say on the history and treatment of the fever.

1. The pauper immigrants from Ireland, are its chief victims; but it also affects the Irish pensioners, whose means must have kept them above the minimum of diet to which the former had been reduced by the famine; finally, it invades the officers and seamen of the ships which bring them over, and the physicians and nurses who wait upon them after their arrival. A great number die on the voyages, and many arrive ill; but it has been observed at Gros Isle, that a large number are attacked soon after being landed. Others remain well, and are sent on to Quebec, where a portion of them are taken down, while others escape till they reach Montreal, or the towns above. When at Oswego, in the State of New York, on my way out, I saw a number of cases.

It affects men rather more than women, and adults more than children; hence it has multiplied the number of infants on the banks of the St. Lawrence, to an unprecedented extent. I have already mentioned the mortality of the physicians at Gros Isle, a seventh of whom have died. In the Marine Hospital of Quebec, nine or ten of the old nurses have perished, and others are disabled, so that there is not one now on duty who was there before the fever was introduced. In the sheds, both there and here, but especially there, the crowd of patients is so great, that one, as I have said, can barely turn round among them, and in several of them, men, women and children, are indiscriminately huddled together. As the government has not undertaken to furnish them with clothing, most of them lie in the foul and tattered garments which they wore during the voyage, and perhaps long before. Now whether the disease is propagated by a gas developed chemically, from the organic matter which surrounds them, or by a morbid, aëriform secretion, from their bodies, we are at no loss to account for the sickness of physicians and nurses. On the question of its spread beyond the sheds and hospitals, I have sought for information. The medical gentlemen with whom I have conversed, without a single exception, are of opinion, that it can be communicated by fomites, and cite instances of its appearing in families which had never communicated with any of the wards, but had employed those who had recovered from attacks. In the private hospital of Drs. Douglas and Racey, where there is cleanliness, free ventilation and ample space, very few of the attendants are attacked. On the whole, it appears to me that its mode of propagation should still be kept *sub judice.*

2. After seeing patients in every stage of the fever, and conversing with the gentlemen whom I have named, I may venture to give the following desultory account of its symptoms and progress.

Most of the cases are not seen in the beginning by physicians, and no reliable accounts can be gotten of them; but, on the whole, the majority seem to sicken gradually; and in reference to those who had been greatly reduced by famine, this is perhaps always the case. There are, however, many examples of sudden and violent invasion, followed by a malignant development, and death in a few days. In no instance does the chill become very intense, though it may be protracted; nor is the arterial re-action high. In some cases the latter, in fact, never manifests itself—the vital forces being inadequate to a rally. The pulse is never tense, and in the highest re-action always easily compressible; its frequency is increased, but not to a remarkable degree; it often becomes almost imperceptible, in those who ultimately recover. From the beginning the primæ viæ are more or less, but variously, disordered. In some there is nausea and vomiting; in all, loss of appetite, with thirst. Some are costive in the forming stage and even throughout the fever; in others, there is a *precursory* diarrhœa; in the majority a *supervening* diarrhœa, or actual dysentery. I could not ascertain that there is generally a superabundant excretion of bile. The tongue, at the onset, is always covered with white fur, through which the red papillæ sometimes show themselves; in part the edges and tip of the organ show some unnatural redness, but in the greater number the natural colour is not exalted, but even reduced, so that the white fur seems to shoot out of a pallid membrane. At the same time the organ becomes broader and flatter, loses its elasticity, and receives indentations from the teeth, on which I seldom saw any sordes. Its moisture continues in a remarkable degree; it may be reduced, but not to the point of dryness; and the whiteness of the fur endures to a period equally late. The dry, contracted, mahogany tongue, of genuine typhus, often appears, it is true; but in numerous instances the moist and pale state of the organ continues up to the time of dissolution. The usual inequality of heat, between the upper and lower parts of the body, is common. I saw many patients in which the latter were cold, and some in which the former were decidedly hot, but great development of caloric is not, I think, a constant phenomenon. Delirium is more prevalent than coma. Many patients, during the night, when it is greatest, are restless, and even locomotive—becoming, the next day, composed and of sound mind. Somnolency did not appear to me to be a conspicuous symptom. Headache is often present. Of the dull and red eye I saw much less than I had expected. A circumscribed flush of the cheek is frequent, but not universal. A bilious tinge of the visage occasionally shows itself. Subsultus tendinum is comparatively rare. I saw many who seemed to be almost in *articulo mortis*, and yet showed little or none of that symptom.

The skin shows various kinds of maculæ. In a few, genuine rose-coloured spots show themselves, but very soon assume a darker colour. In the majority, the spots are purple from their first appearance, and of every size, from ordinary petechiæ up to diffused ecchymoses—often bearing a close resemblance to *post-mortem* hyperæmias. In some cases the spots are hard, like whelks, and the seat of a sensation which leads the patient to scratch them, whereupon ulcers follow, which occasionally assume a sloughing character. Hæmorrhages from the nose are somewhat common, from the bowels and skin not quite so frequent; nevertheless, all the medical gentlemen have had cases of well-marked purpura hæmorrhagica, mixed up with the fever cases; and it may be safely affirmed, that in these immigrants, the blood, under the influence of a reduced or unhealthy diet, has become signally deteriorated.

When the fever assumes a protracted form, anasarcous infiltrations into the cellular tissue of the lower extremities or the face, frequently take place. Suppurations, in addition to those of the skin just mentioned, are common. Those about the back and hips, may be ascribed to pressure; but others, occurring in glands, must be referred to the fever. Of these organs, the parotids suffer oftener than all the rest, and the discharge of pus, when they suppurate, is copious; such cases generally end well.

A supervening bronchial or pulmonary affection, is, on the other hand, ominous, and, as it frequently occurs, may be considered one of the modes in which the fever comes to a fatal termination.

But of all the secondary affections, that of the bowels is most frequent and fatal; though death may not occur for a considerable time after the febrile period has expired. This intestinal disorder seems to be a sort of mixed up diarrhœa and dysentery, under which the patient loses the original febrile symptoms, and, becoming extremely emaciated, gradually sinks. In some instances, this affection sets in during the fever—in others it is excited, in the period of convalescence, by irregularities of diet: in all, it is an ugly, obstinate, and unmanageable *addendum*. In the months of June and July, it was much less frequent than at the present time, when so large a proportion of the patients labour under it, as almost to constitute it a new act in the melancholy drama.

I have mentioned the nocturnal delirium of some patients, indicating an exacerbation at night; and may add to this evidence of periodicity, that in a few cases there has been a diurnal recurrence of the initial chilliness; the general character of the fever, however, is continued. I have spoken of cases which prove fatal in three or four days. They are few in number; and the common duration is from two to three weeks, always excepting those which merge in diarrhœa or dysentery, when the end is quite indefinite.

When death is the consequence of cerebral pulmonary, hepatic or intestinal concentration, the reason of its occurrence is intelligible enough; but the majority do not seem to die from these lesions; and the cause of their dissolution is, *prima facie*, rather obscure. In every ward that I visited, I was surprised at the small amount of visible manifestation of dangerous disease; and, more than once, was prompted to say to the medical gentlemen, "I can't see why so many of your patients die." In wards from which many corpses were daily carried out, there would be but few who did not look at and after us; put out their moist tongues with facility, and make known their wants; yet many such patients die soon afterwards. Others die, when the physician has pronounced them convalescent—others after they have risen and dressed themselves, and crawled into the open air. Such deaths cannot be regarded as the effect of any particular organic lesion, but of a stage of exhaustion or collapse, bearing some resemblance to the third stage of yellow fever, or the recurring chill of a malignant intermittent in the South West; but more, perhaps, to the fatal stage of epidemic cholera.

After this imperfect, but, I believe, not unfasthful sketch of the history of this fever, I must proceed to its cure.

3. It is a maxim with the physicians of Gros Isle and Quebec, that a great amount of medication is inadmissible in this malady. Scarcely a patient is ever brought to them while the fever is still in its forming stage, so that there are but few opportunities of knowing whether art could arrest it in that stage. Once established in the system, the general opinion is that it cannot be cut short. The treatment, then, is merely palliative and corroborative.

By all the physicians, in almost every case, the lancet is repudiated, even when the patients are commanders of ships, and seamen, in whom famine had not preceded the attack. Local bleeding is almost as little employed, the majority of the physicians preferring counter-irritants, when the brain or the lungs are affected.

Emetics are prescribed by some, who speak well of their effects; but others think they predispose to congestions of the brain. It has not been observed by any that they break up the disease.

All employ cathartics; but the kinds, and the degree of their administration, present considerable variety; into which, however, I shall not enter, as drastic or long-continued purging is condemned by the whole.

Of sweating I can say nothing, for the patients, generally, are placed under such circumstances as preclude a resort to it.

A standard, or standing, febrifuge mixture, at Gros Isle, as given me by the apothecary, Mr.Barter, is the following :—R. Sod. et pot. tart., ℥ij.; liq. ammon. acet., ℥jss.; spt. ether, nitr., ℥ss.; aquæ commun., ℥xij. Misce. Another is composed of the camphorated mixture, tincture of hyoscyamus and tartar emetic.

Some of the medical gentlemen attach but little value to this class of medicines, and rely, during the more acute stages of the fever, chiefly on free dilution; but advise against the addition of acids, as likely to irritate the bowels into diarrhœa.

Opium is not in much favour in any stage of the fever. At a comparatively early period, some physicians commence the administration of wine and aliment, a practice condemned by others, as dangerous to the brain; but all concur in this, that sooner or later, and sometimes quite early, there comes a pathological condition which demands prompt and energetic stimulation. In this resort a choice of means is not to be neglected. Of the whole materia medica, camphor is the most reliable. It is given in doses of ten, twenty or even thirty grains, and often arrests the sudden sinking of the powers of life, and determines a speedy recovery. At the same time alcoholic stimulants, sulphate of quinia, food and sinapsims are employed.

For the diarrhœa and dysentery, the cretaceous mixture, hydrarg. cum creta, astringents, and diluted nitric acid, with laudanum, are the usual prescriptions.

MONTREAL, September 3d.

A large number are sick in the sheds at this place; attacked, of course, after they had left the quarantine ground. A great proportion of them are said to have disentery. The deaths are numerous. The fever, here, is by no means confined to immigrants, but has invaded the city, and added greatly to its ordinary summer mortality. Its victims, however, are largely of that class which, living near the wharves, received and mingled with the immigrants, before sheds were provided for their reception. In conversing with several of the most intelligent physicians here, I find the treatment to be substantially the same as at Gros Isle and Quebec; but latterly, as Dr. Badgely informed me, increasing reliance is placed on nitric acid. He uses the following formula:—℞. Acid nitr. ʒj.; alcohol, ℥iv.; aquæ ℥iv. Misce. An ounce is to be given every hour, beginning early in the disease, and without much preparation of the system. Under its administration, he and other gentlemen have, as he assured me, seen the pulse rapidly reduced in frequency, with a corresponding abatement of all the febrile symptoms.

When at Gros Isle I inquired as to *post-mortem* inspections, and could hear of only two. They were made by Dr. Watt, who found the liver engorged, and, as he believed, fatty. The state of the organs convinced him that the one mentioned suffered more than any other, which led him to prescribe purges of calomel and gamboge. In Quebec I could not learn that any dissections had been made. In this city Dr. Fraser has published, in the July No. of Dr. Hall's valuable "British American Journal of Medical and Physical Sciences," a short paper, in which he says:—

"The morbid appearances found on dissection, are venous congestion, with effusion of serum on the surface, in the ventricles, and base of the brain, but no trace of active inflammation. When the case has been complicated with bronchitis, I have found the bronchial mucous membrane throughout, tumid, swollen, highly vascular, and containing much mucus; the vascularity extending to the submucous tissue, with congestion and partial hepatization of portions of the lungs. When diarrhœa has existed, the small intestines, especially the lower portion of the ileum, has presented the appearance of active congestion of its mucous coat, which was slightly thickened, without being softened; some patches had the appearance of sanguineous extravasation, not unlike the maculæ observed on the skin. When the patient had a jaundiced appearance, a common occurrence in this epidemic, I have found the liver enlarged from congestion, presenting a bloody and bilious appearance when cut into, and the gall-bladder distended with inspissated bile, thick enough to maintain its form when deprived of its covering. When there has been only a slight bilious tinge of the skin and conjunctivæ, the liver presented the same appearance in a less degree, the bile in the gall-bladder being about the consistence of treacle."

Dr. Fraser does not tell us how many autopsies he had made; and we cannot but regret that so little has been done, where the opportunities are so great. It may be given in extenuation, however, that the physicians in attendance are overworked, and that many of them have been ill with the fever.

I have no time to give you the statistics of this disease, had I been able to collect full data. The number of deaths, in proportion to that of the cases, never will be known, as nearly every form of disease in the immigrants goes very much under one name. I may say, in general terms, that the mortality has been, and still continues, very great. Take the following as specimens. At Gros Isle, for the week preceding the 24th of August, the number of patients averaged more than 2,000, and the deaths amounted to 288. In the Marine and Emigrant Hospital of Quebec, the average for the week ending the 21st of August was about 850, the deaths 166; to which might be added 34 said to have died of dysentery, a sequel of the fever. The Montreal returns would be about the same; and present, in the aggregate, about 100 deaths a day at the three places. At Gros Isle alone, up to the 20th of August, the deaths had amounted to 2116. The disease had then prevailed about three months. A portion of them, however, have resulted from smallpox, which has prevailed to a considerable extent on the island, and more or less, I believe, in Quebec and Montreal, certainly in the latter, where I saw it in the General Hospital.

The British government have lately made an effort to arrest the fever by disinfecting agents. Dr. Stratton, of the Royal Navy, but sojourning in Upper Canada, has been commissioned to this enterprise, and arrived in Quebec while I was there. He was advised that two agents would be forwarded to him. One proved, on opening the package, to be Sir William Burnett's patent disinfecting fluid; the other, a small bale of dresses for a lady! which had been forwarded from Halifax (by mistake) instead of Ledoyen's disinfecting fluid. The former (of which Dr. Stratton kindly presented me with a small vial, which I carry in my trunk as an amulet) is said by the medical gentlemen of this quarter, to be a solution of the chloride of zinc. The latter, according to Dr. Hall, is a liquor of nitrate of lead. When I left Quebec, arrangements were making for experiments in the Marine Hospital, but its physicians seemed to think lightly of the practical value of such measures. When I was at Gros Isle, Dr. Douglas, the Quarantine Physician, sagaciously remarked, that the proper place for such experiments is an emigrant ship on her voyage.

In conclusion, I must beg of you to correct at least the grosser blunders of style, which are unavoidable in the circumstances under which I have written this most hurried epistle. With all its imperfections, it may, however, be of some interest to such of your readers as may not have seen much of what may have have been written by others on the Irish epidemic; and in that conviction I dismiss it, by subscribing myself, very respectfully, Your ob't servant,

DAN. DRAKE.

